# The Mayo Hospital, Rochester, U.S.A.

**Published:** 1913-04-19

**Authors:** 


### THE MAYO HOSPITAL, ROCHESTER, U.S.A.

### A Wonderful Example of Specialisation.

For the last decade or so every surgeon visiting the United States has made a point of going to see the Mayo Clinic at Rochester, Minnesota. This unique organisation might justly be called the Mecca of the surgical world at the present date; and it certainly attracts pilgrims from even more diverse regions of the earth than the famous Arabian city does. As a school of advanced surgery it has been many times described by British surgeons who have been there; and it is no part of our present purpose to dwell upon the surgical side of the oft-told tale, except in so far as is necessary to explain and illustrate the institutional aspect of the extraordinary hospital there.

### The Mecca of Surgery.

Rochester, it may be premised, is quite a small town, hardly more than a big village, in Minnesota. It is not on any main railway line; and but for its hospital it is safe to say that its name would be quite unknown to the citizens of the United States. In this out-of-the-way spot two surgeons, the brothers Mayo, have built up a private practice in operative surgery; and that practice is, beyond all doubt, many times larger than any other in the world. They have a private hospital of three hundred beds, in which an average of thirty operations a day are performed. This means an average stay of ten days per patient—a record which is rendered possible by the custom of turning over all semi-convalescents to the large hotels in Rochester as soon as it is safe to have them removed. The gross receipts of this undertaking have been estimated at half a million a year; from this, of course, enormous expenses have to be deducted before the net profits can be calculated. No two men could without help conduct surgery on so extensive a scale, not even the Mayos: the organisation by which they cope with the work is the most striking instance of specialisation with which we are acquainted.

Let us follow the adventures of a patient who has been advised to undergo operation, and has decided to put himself into the Mayo Hospital for the purpose. He makes his way to Rochester on a special patients' train, and engages a room at an hotel. Then he interviews a business manager, to whom he has to disclose his financial circumstances; upon these he is assessed at a certain sum, which he must pay for his treatment. The very wealthy pay, it is commonly bruited and believed, very large fees; those of comparatively small means are assessed at very moderate rates. After that, the patient attends an "office," where an assistant takes down his medical history in detail. He is then subjected to such examinations as may seem necessary to elucidate the diagnosis at the hands of various specialists and experts on the staff of the hospital. One man, for instance, does nothing but test-meal examinations year in and year out, at which he naturally becomes highly expert. Another sticks to cystoscopy, catheterisation of the ureters, and skiagraphy of the renal pelvis after filling it with collargol. This refinement of diagnosis in renal surgery is quite a modern, almost a new, development, and the Mayo Clinic is similarly equipped for every other novel aid to efficiency which has been proved to be valuable. There are about thirty of these expert specialists attached to the hospital.

When the case has been thus probed, as thoroughly as seems necessary and possible, by one or more of this expert staff, the patient returns to his hotel, where he awaits a notification that there is a vacant bed for him in the hospital. On admission the notes of his case, with the results of all the investigations which have been conducted, are laid before one of the operators, who decides whether an operation, and if so what, is to be performed. The operators are five in number: the two Mayos, and three others. William Mayo does abdominal surgery almost exclusively. His brother Charles makes a speciality of goitre, but does, of course, other work as well; and the three remaining members of the staff also have special lines of surgical work. All five are absolutely at the top of the profession in America; they have done and are doing valuable original work in the magnificent clinic which provides them with such excellent materials for research.

### The Theatre Units.

It has been mentioned above that some thirty operations a day are performed, an average of six to each operator. Needless to say, each surgeon has a separate theatre unit to himself. Work begins at eight o'clock sharp in the morning, and goes on as a rule to about lunch time. The anæsthetics are given by nurses, not by qualified doctors; open ether alone is administered, and those who have visited Rochester bear unanimous testimony to the excellence with which the anæsthesia is induced and conducted. A great many visitors are present at the operations, and special arrangements are made for their comfort and convenience, and to combine that with the entire freedom of the operator and his assistants. There is a special "Surgeons' Club" for these professional visitors, and many unusual provisions for making their stay profitable and pleasant; but, since these are somewhat apart from the institutional aspects of the Mayo Hospital, we need not go into details here. The operating surgeons teach throughout their morning's work, especially the elder Mayo.

It has been mentioned that semi-convalescents are discharged to their hotels at the earliest practicable moment. This course results in a short average stay of each patient in hospital, and renders possible the enormous amount of work which is done. Nor is it a measure which need alarm the nervous; for the hotels of Rochester have grown up, as it were, round the Mayo Hospital, and are well equipped for the purpose of caring for those recovering from operations. That the system works well is sufficiently shown by its marvellous success, and by the perpetual demand for admission to the hospital, which is always full.

Institutional Management in Private Practice.

In all essentials, the Mayo Hospital is the application to private practice of the principles of institutional management. Here is a three-hundred-bed hospital, complete with an out-patient and an in-patient staff, and with special departments for dealing with clinical pathology, regional specialisation, and the rest. The main difference is that this is a private institution for paying patients; though, even then, it includes an organisation which has distinct resemblances to the almoner's office. There is no lack of funds for carrying on the work, and, in consequence, it is possible to offer adequate salaries to the junior medical officers, and so to command their exclusive services. Thus it is feasible to push to its logical development the principle of specialisation; while at the same time the specialist is kept under due control by the fact of treatment remaining in the hands of someone else. It may be, indeed it surely must be, that to spend every day and all day analysing vomit, counting blood corpuscles, or in any similar section of clinical pathology, is not exactly a stimulating or amusing prospect. But it must make for efficiency, and there is unanimous testimony that at Rochester it does so.

The inevitable reflection, when one considers the lessons of this gigantic organisation, is this: If one man (or one firm) can evolve such a system in America, why should not another do the same elsewhere? The thing has been done once, improbable and impossible as the feat would have been thought a few years ago; to follow this example seems obvious enough as a road to fame and affluence. One of the latest of the returned visitors to Rochester who have put their experiences into print * supplies a reason, which is, doubtless, the correct explanation of the lack of imitators, or of successful imitators, of the Mayo Hospital. William Mayo, he says, is not merely one of the foremost abdominal surgeons of the world. He is something much more uncommon even than a really great surgeon. He has also, besides surgical abilities which are unquestionably remarkable, that talent for organisation, for selection of his subordinates, for supervision, and that indomitable industry which together mean genius of the Napoleonic order. Had William Mayo, says Mr. Back, taken to politics, he would by now have been President of the United States; whatever walk of life he had chosen, he would have got to the head of it. He chose surgery, and the Mayo Hospital is the result. Furthermore, according to the same author, when this extraordinary man retires from the enterprise its days are numbered. William Mayo is the driving force which keeps the whole intricate mechanism going, just as he is the man who first built it up. There is, one may suppose, the possibility that a man of equal talents and personality may be found to succeed him; but Napoleons are not to be found every day, and Mr. Back's prophecy may quite well turn out to be an accurate one. Whether this is so or not, at least the Mayo Hospital can claim to-day a place in the institutional world without a parallel and without a rival; and the United States most certainly contains the "biggest thing on airth" in private hospitals and in private surgical practices. Not the least of the marvels connected with it is the fact that it should have been created and nurtured not in one of the great cities of America, but in an out-of-the-way place which is scarcely large enough to merit the name of a town.

* Ivor Back, F.R.C.S., *St. George's Hospital Gazette*, March, 1913.

